# SARS-CoV-2 triggers pericyte-mediated cerebral capillary constriction

**DOI:** 10.1093/brain/awac272

**Published:** 2022-07-22

**Authors:** Chanawee Hirunpattarasilp, Greg James, Jaturon Kwanthongdee, Felipe Freitas, Jiandong Huo, Huma Sethi, Josef T Kittler, Raymond J Owens, Laura E McCoy, David Attwell

**Affiliations:** Department of Neuroscience, Physiology & Pharmacology, University College London, London, WC1E 6BT, UK; Princess Srisavangavadhana College of Medicine, Chulabhorn Royal Academy, Talat Bang Khen, Lak Si, Bangkok 10210, Thailand; Department of Neuroscience, Physiology & Pharmacology, University College London, London, WC1E 6BT, UK; Department of Neurosurgery, Great Ormond Street Hospital, London, WC1N 3JH, UK; Department of Neuroscience, Physiology & Pharmacology, University College London, London, WC1E 6BT, UK; Princess Srisavangavadhana College of Medicine, Chulabhorn Royal Academy, Talat Bang Khen, Lak Si, Bangkok 10210, Thailand; Department of Neuroscience, Physiology & Pharmacology, University College London, London, WC1E 6BT, UK; Division of Structural Biology, Nuffield Department of Medicine, University of Oxford, OX3 7BN, UK; Protein Production UK, The Research Complex at Harwell, and Rosalind Franklin Institute, Harwell Science & Innovation Campus, Didcot, OX11 0GD, UK; National Hospital for Neurology and Neurosurgery, Queen Square, London, WC1N 3BG, UK; Department of Neuroscience, Physiology & Pharmacology, University College London, London, WC1E 6BT, UK; Division of Structural Biology, Nuffield Department of Medicine, University of Oxford, OX3 7BN, UK; Protein Production UK, The Research Complex at Harwell, and Rosalind Franklin Institute, Harwell Science & Innovation Campus, Didcot, OX11 0GD, UK; Division of Infection and Immunity, University College London, London NW3 2PP, UK; Department of Neuroscience, Physiology & Pharmacology, University College London, London, WC1E 6BT, UK

**Keywords:** pericyte, SARS-CoV-2, capillary

## Abstract

The SARS-CoV-2 receptor, ACE2, is found on pericytes, contractile cells enwrapping capillaries that regulate brain, heart and kidney blood flow. ACE2 converts vasoconstricting angiotensin II into vasodilating angiotensin-(1-7). In brain slices from hamster, which has an ACE2 sequence similar to human ACE2, angiotensin II evoked a small pericyte-mediated capillary constriction via AT1 receptors, but evoked a large constriction when the SARS-CoV-2 receptor binding domain (RBD, original Wuhan variant) was present. A mutated non-binding RBD did not potentiate constriction. A similar RBD-potentiated capillary constriction occurred in human cortical slices, and was evoked in hamster brain slices by pseudotyped virions expressing SARS-CoV-2 spike protein. This constriction reflects an RBD-induced decrease in the conversion of angiotensin II to angiotensin-(1-7) mediated by removal of ACE2 from the cell surface membrane, and was mimicked by blocking ACE2. The clinically-used drug losartan inhibited the RBD-potentiated constriction. Thus, AT1 receptor blockers could be protective in Covid-19 by preventing pericyte-mediated blood flow reductions in the brain, and perhaps the heart and kidney.

### Introduction

Despite the primary site of infection by SARS-CoV-2 being the respiratory tract, the virus evokes dysfunction of many other organs, including the brain, heart and kidney: 36% of hospitalised patients show neurological symptoms,[1] 20% develop myocardial injury[2] and 41% experience acute kidney injury.[3] This could reflect either a spread of virus via the blood,[4] or the effects of inflammatory mediators released from the lungs. These effects may contribute to "long Covid", in which clouding of thought and physical exhaustion extend for months after the initial infection.

The receptor[5,6] for SARS-CoV-2 is the enzyme angiotensin converting enzyme 2 (ACE2, part of the renin-angiotensin system that regulates blood pressure), which converts[7] vasoconstricting angiotensin II into vasodilating angiotensin-(1-7). The Spike protein of SARS-CoV-2 binds to ACE2 to trigger its endocytosis.[6] For the closely-related SARS virus, binding of only the receptor binding domain (RBD) is sufficient[8] to evoke internalisation of ACE2.

In the heart[9] and brain[10,11] the main cells expressing ACE2 are pericytes enwrapping capillaries (with some expression in endothelial cells), and pancreas and lung pericytes also express ACE2.[12,13] Pericytes express contractile proteins and in pathological conditions have been shown to constrict capillaries and decrease blood flow in the brain,[14,15] heart[16] and kidney.[17] Interestingly, a decrease of blood flow has been reported for SARS-CoV-2 infection in the brain[18-20] and kidney.[21] One brain study[18] was a single case report that used arterial spin label (ASL) and dynamic susceptibility contrast magnetic resonance imaging (MRI) techniques to show an asymmetric marked reduction of cerebral blood flow in the bilateral fronto-parietal regions. A further perfusion imaging study of 11 patients[19] found bilateral frontotemporal hypoperfusion in all of them. Another ASL MRI study[20] of 51 patients who had recovered from Covid-19 showed that patients who had severe disease suffered from a prolonged and widespread decrease of cerebral blood flow.

Since pericytes have been reported to be infected by SARS-CoV-2 in Covid-19,[11] these blood flow reductions could be due to pericyte dysfunction caused by SARS-CoV-2 reducing the activity of ACE2, either by occluding its binding site for angiotensin II (although this is thought not to occur either for the related SARS virus[22] or for SARS-CoV-2)[23,24] or by promoting removal of the enzyme from the membrane.[6,8] In the presence of angiotensin II (either renally-derived and reaching the brain parenchyma via a compromised blood-brain barrier, or generated by the brain's own renin-angiotensin system),[25] a reduction of ACE2 activity would increase the concentration of vasoconstricting angiotensin II and decrease the concentration of vasodilating angiotensin-(1-7). We therefore investigated the effect of the SARS-CoV-2 RBD and Spike protein on the control of capillary diameter by pericytes.

## Materials and methods

**Animals** Brain slices (200-300 μm thick) were made from the brains of Syrian golden hamsters (age 5-24 weeks) of both sexes, which were humanely killed (in accordance with UK and EU law) by cervical dislocation after being anaesthetised with isoflurane. In each slice only one pericyte was studied. The constriction evoked by angiotensin II in the presence of the RBD showed overlapping ranges of value for 2 female vessels and 9 male vessels (not significantly different, p=0.67).

**Imaging of pericyte mediated constriction** Pericytes on cortical capillaries were identified visually as previously described (Fig. S1 of ref. 15, and see below) and imaged with a CCD camera as described.[26] Diameter was measured in Metamorph software (Molecular Devices) by drawing a line across the vessel between the inner walls of the endothelial cells.

**RBD and mutant RBD synthesis** Codon optimised Genblocks (IDT Technology) for the receptor binding domain (RBD amino acids 330-532) of SARS-CoV-2 (original Wuhan variant; Genbank MN908947) and human Angiotensin Converting Enzyme 2 (ACE-2, amino acids 19-615) were inserted into the vector pOPINTTGneo (PMID: 25447866) incorporating a C-terminal BirA-His6 tag and pOPINTTGneo-3C-Fc to make C-terminal fusions to Human IgG Fc. The (non-binding) RBD-Y489R mutant was generated by firstly amplifying the RBD-WT gene using oligos TTGneo_RBD_F and RBD-Y489R_R, as well as RBD-Y489R_F and TTGneo_RBD_R; followed by joining the two resulted fragments with TTGneo_RBD_F and TTGneo_RBD_R.

| | |
|---|---|
| TTGneo_RBD_F | 5'-gcgtagctgaaaccggcccgaatatcacaaatctttgt-3' |
| TTGneo_RBD_R | 5'-GTGATGGTGATGTTTATTTGTACTTTTTTTCGGTCCGCACAC-3' |
| RBD-Y489R_F | 5'-GGCGTCGAGGGTTTTAACTGTCGCTTCCCACTTCAGTCATACGG-3' |
| RBD-Y489R_R | 5'-CCGTATGACTGAAGTGGGAAGCGACAGTTAAAACCCTCGACGCC-3' |

The gene carrying the Y489R mutation was then inserted into the vector pOPINTTGneo incorporating a C-terminal His6 tag by Infusion® cloning. The plasmid was sequenced to confirm that the mutation had been introduced successfully. Recombinant protein was transiently expressed in Expi293™ (ThermoFisher Scientific, UK) and purified from culture supernatants by immobilised metal affinity chromatography using an automated protocol implemented on an ÄKTAxpress (GE Healthcare, UK) followed by a Superdex 200 10/300GL column, using phosphate-buffered saline (PBS) pH 7.4 buffer. Recombinant RBD-WT and ACE2-Fc were produced as described.[27] The sequence of the RBD was:

*ETG*P**N**ITNLCPFGEVF**N**ATRFASVYAWNRKRISNCVADYSVLYNSASFSTFKCYGVSPTK
LNDLCFTNVYADSFVIRGDEVRQIAPGQTGKIADYNYKLPDDFTGCVIAWNSNNLDSKV
GGNYNYLYRLFRKSNLKPFERDISTEIYQAGSTPCNGVEGFNCYFPLQSYGFQPTNGVG
YQPYRVVVLSFELLHAPATVCGPKKSTN*KHHHHHH*

where the residues in italics are derived from the expression vector. Glycosylated residues are shown in bold (**N**) and the tyrosine that is mutated to arginine (Y489R) in the mutant RBD is shown underlined.

**Surface plasmon resonance** Experiments were performed using a Biacore T200 system (GE Healthcare). All assays were performed using a Sensor Chip Protein A (GE Healthcare), with a running buffer of PBS pH 7.4, supplemented with 0.005% vol/vol surfactant P20 (GE Healthcare), at 25 °C. ACE2-Fc was immobilized onto the sample flow cell of the sensor chip; the reference flow cell was left blank. RBD-WT or RBD-Y489R (0.1 μM) was injected over the two flow cells, at a flow rate of 30 μl min−1 with an association time of 60 s.

**Solutions** Brain slices were superfused at 33-36$^{o}$C with solution containing (mM): 124 NaCl, 2.5 KCl, 1 $MgCl_2$, 2 $CaCl_2$, 1 $NaH2PO_4$, 26 $NaHCO_3$, 10 D-glucose and 0.1 ascorbate, bubbled with 20% $O_2$/70% $N_2$/5% $CO_2$ to ensure a physiological [$O_2$] was achieved in the slice[14]. The high molecular weight of the RBD (~31 kD) implies it will not diffuse rapidly from the superfusion solution into brain slices so, to apply the RBD, we pre-incubated each slice in solution containing RBD (at 35$^{o}$C, to allow time for diffusion) prior to placing the slice in the imaging chamber, where it was superfused with the same solution containing the RBD. This 30 min pre-incubation time was mimicked for slices that RBD was not applied to. The same procedure was followed for the mutant RBD and for pseudovirus application.

**Immunohistochemistry** Hamster brain slices were fixed in 4% paraformaldehyde (PFA) while shaking at room temperature for 20 min (except for experiments with pseudotyped virions; 1 hour) and washed 3 times in phosphate-buffered saline (PBS). For detection of ACE2, antigen retrieval using sodium citrate buffer (consisting of 10 mM sodium citrate, 0.05% Tween 20 and HCl to adjust the pH to 6.0) for 20 min was performed and the slices were left to cool down for 20 min before being washed in PBS for 5 minutes. Brain slices with or without antigen retrieval were transferred to blocking solution containing 10% horse serum, 0.2% saponin (Sigma-Aldrich, S7900 for ACE2 detection) or 0.3% Triton X-100, 200 mM glycine and 150 μM bovine serum albumin in PBS at $4^{o}$C, and shaken for 4 hours at room temperature or overnight at $4^{o}$C for ACE2 detection. Slices were incubated in the blocking solution with primary antibodies for 24 hours (72 hours for ACE2 detection) at $4^{o}$C with agitation, washed with PBS 4 times, incubated with the secondary antibody overnight at 4°C with agitation and washed again 4 times with PBS. For nucleus counter staining, slices were incubated in PBS containing DAPI (100 ng/ml) for 1 h at room temperature and washed in PBS for 5 min. Primary antibodies used were goat anti-ACE2 (R&D systems, AF933, 1:200), goat anti-CD31 (R&D systems, AF3628, 1:200), goat anti-PDGFRβ (R&D Systems, AF385, 1:200), mouse anti-NG2 (Abcam, ab50009, 1:200), rabbit anti-PDGFRβ (Santa Cruz, sc-432, 1:200) and rabbit anti-angiotensin II type 1 receptor (Abcam, ab124505, 1:100). Secondary antibodies used were Alexa fluor 488 donkey anti-goat (Invitrogen, A11055, 1:500), Alexa fluor 555 donkey anti-mouse (Invitrogen, A31570, 1:500) and Alexa fluor 647 donkey anti-rabbit (Invitrogen, A31573, 1:500).

**Pericyte identification** Pericytes were identified morphologically (pericytes are located on the outside of capillaries with their nuclei showing a "bump on a log" morphology, and at the intersection of capillary branches), when visualised through staining either the basement membrane with isolectin B4 (IB4) or the pericyte cell membrane using anti-PDGFRβ or anti-NG2 antibodies (Supp. Fig. 1).[28] Pericytes are completely embedded in (encircled by) the basement membrane, which in hamsters can be labelled with IB4 conjugated to Alexa Fluor 647 (Invitrogen, I32450, 3.3 μg/ml, applied for 30 mins before fixation and subsequent immunohistochemistry [in rats and mice IB4 also works when applied with the secondary antibodies during immunohistochemistry]). In contrast, a smaller population of perivascular cells that expresses PDGFRβ like pericytes are fibroblasts, which have a flatter soma and are outside the basement membrane and so show IB4 labelling only on the capillary side of the cell.[29,30] We found that out of 30 PDGFRβ expressing peri-capillary cells, 93.3% were completely surrounded by IB4 and hence were pericytes. We have also previously shown that identifying pericytes morphologically gives excellent agreement with identification based on IB4 labelling (see Supp. Fig. S1 of ref. 15).

**Pericyte death assessment** Brain slices (300 μm thick) were incubated for 3 h at 35$^o$C in extracellular solution (bubbled with 20% $O_2$, 5% $CO_2$ and 75% $N_2$) containing 7.5 μM propidium iodide (PI; Sigma-Aldrich, 81845) and IB4 conjugated to Alexa Fluor 647, with and without RBD (0.7 mg/l) and/or angiotensin II (50 nM). The slices were fixed with 4% PFA for 1 hour and washed 3 times with PBS, for 10 minutes each time. Nucleus counterstaining was achieved by incubating the slices in PBS containing DAPI (100 ng/ml) for 1 hour, washing 1 time with PBS. Imaging of Z stacks (approximately 320 μm x 320 μm x 20 μm) was performed on a confocal microscope. The first 20 μm from the surface were discarded to exclude cells killed by the slicing procedure.

**Human tissue** Live human cerebral cortical tissue was obtained from the National Hospital for Neurology and Neurosurgery (Queen Square, London). Tissue was taken from female subjects aged 40–74 undergoing tumour resection. Healthy brain tissue overlying the tumour, which had to be removed for the operation and which would otherwise have been discarded, was used and was transported to the lab in less than 30 mins at 1-5$^o$C. Ethical approval was obtained (NHS REC North-Western board: REC number 15/NW/0568, IRAS ID 180727 (v3.0), "Properties of human pericytes", as approved on 9-10-2018 for extension and amendment) and all patients gave informed written consent. All tissue handling and storage were in accordance with the Human Tissue Act (2004).

**Quantification of ACE2 expression on the pericyte surface** Brain slices (200 μm thick) were incubated in extracellular solution gassed with 20% $O_2$, 5% $CO_2$ and 75% $N_2$, with and without RBD (0.7 mg/l) for 3 hours. Immunohistochemistry was performed to detect ACE2 and PDGFRβ expression. Images of randomly selected pericytes (approximately 39 μm x 39 μm) were taken using a confocal microscope. For each image, a mask of PDGFRβ, which is expressed on the cell membrane, was created and the mean fluorescence intensity representing ACE2 expression within that membrane area was measured (isolated puncta in the PDGFRβ image, located away from pericytes, were digitally removed). For the soma the ACE2 intensity in the intracellular space bounded by the membrane (defined as described) was also measured.

**SARS-CoV-2 pseudotyped virion production** HIV-1 particles pseudotyped with SARS-CoV-2 spike were made as previously described.[31] Briefly, a T75 flask was seeded the day before with 3

million HEK293T/17 cells in 10 ml complete DMEM, supplemented with 10% FBS, 100 IU/ml penicillin and 100 μg/ml streptomycin. Cells were transfected using 60 μg of PEI-Max (Polysciences) with a mix of three plasmids: 9.1 μg HIV-1 luciferase reporter vector[32], 9.1 μg HIV p8.91 packaging construct and 1.4 μg WT SARS-CoV-2 Spike expression vector[32]. Supernatants containing pseudotyped virions were harvested 48 h post-transfection, filtered through a 0.45-μm filter. Infectivity was titrated by serial dilution of supernatant in DMEM (10% FBS and 1% penicillin–streptomycin) followed by addition to HeLa cells (10,000 cells per 100 μl per well) that stably express ACE-2 (provided by J.E. Voss, Scripps Institute). After 48-72 h luminescence was assessed as a proxy of infection by lysing cells with the Bright-Glo luciferase kit (Promega), using a Glomax plate reader (Promega).

**Pseudotyped virion application** Brain slices (300 μm thick) were incubated in extracellular solution containing either pseudotyped virions (8375 TCID50/ml final concentration when applied at a 1:10 dilution from the harvested viral supernatant) or DMEM (1:10; as a control), oxygenated with 20% $O_2$, 5% $CO_2$ and 75% $N_2$ for 30 min. Angiotensin II (50 nM) was added to the solution and then slices were incubated for another 30 min. The slices were fixed with 4% PFA for 1 hour to inactivate the virions. Immunohistochemistry for CD31 and PDGFRβ with nuclear counterstaining was performed and the slices were imaged as Z stacks (~ 320 μm x 320 μm x 16 μm) with a confocal microscope. Pericyte somata were identified by expression of PDGFRβ and DAPI. The capillary diameter was measured at each pericyte soma and 5, 10 and 15 μm away from the soma[15], by drawing a line across the vessel between the outer walls of the endothelial cells (as defined by the CD31 signal).

**Statistics** Data are presented as mean±s.e.m. averaged over pericytes (the responses of which show more variance than that between animals) or image stacks; number of animals from which the data were taken are given in the figure legends. Experiments using drugs were interleaved randomly with control experiments lacking drugs. For bar graphs individual data points are superimposed on the mean data. Data normality was assessed with Shapiro-Wilk or D'Agostino-Pearson omnibus tests. Comparisons of normally distributed data were made using 2-tailed Student's t-tests. Equality of variance was assessed with an F test, and heteroscedastic t-tests were used if needed. Data that were not normally distributed were analysed with Mann-Whitney tests. P values were corrected for multiple comparisons using a procedure equivalent to the Holm-Bonferroni method (for N comparisons, the most significant p value is multiplied by N, the 2nd most significant by N-1, the 3rd most significant by N-2, etc.; corrected p values are significant if they are less than 0.05).

**Data availability**

Data plotted in the figures are available in an uploaded supplementary file.

**Code availability**

No custom code was used in this manuscript.

## Results

***Angiotensin II evokes pericyte-mediated capillary constriction via AT1 receptors***

To study the effect of the SARS-CoV-2 RBD on cerebral capillary pericyte function, we employed live imaging[26] of brain slices from Syrian golden hamsters. Hamsters have an ACE2 sequence, in the part of the protein that binds the SARS-CoV-2 Spike protein, which is more similar to that in humans than is the rat and mouse ACE2 sequence.[33] In particular amino acid 353 in hamsters and humans is a lysine (K) rather than a histidine (H), and this is a key determinant[34] of how well coronaviruses bind to ACE2, making hamsters a good model for studying SARS-CoV-2 effects.[33]

We assessed the location of ACE2 and contractile properties of pericytes in the cerebral microvasculature of the hamster, which have not been studied previously (Fig. 1). Immunohistochemistry (IHC) revealed ACE2 to be predominantly expressed in capillary pericytes expressing NG2 and PDGFRβ (Fig. 1A, B). Quantification of overlap with the pericyte marker PDGFRβ revealed ~75% co-localisation (Fig. 1C), and comparison of expression in capillaries and penetrating arterioles showed that capillaries exhibited ~75% of the ACE2 expression (Fig. 1D). These results are consistent with transcriptome and IHC data from mouse and human brain[10,11] and human heart[9]. As for brain pericytes in rats,[35] the thromboxane $A_2$ analogue U46619 (200 nM) evoked a pericyte-mediated capillary constriction and superimposed glutamate evoked a dilation (Fig. 1E).

Applying angiotensin II (150 nM) evoked a transient constriction, which was inhibited by the AT1 receptor blocker losartan (20 μM, Fig. 1F). Immunohistochemistry revealed the presence of AT1 receptors on capillary pericytes, as well as on other cortical cells (Supp. Fig. 1A-B). Similar angiotensin-evoked pericyte-mediated capillary constriction has been reported in the kidney[36]

(where the angiotensin receptors were shown to be on the pericytes themselves[37]) and retina[38] (where angiotensin evokes a rise in $[Ca^{2+}]_i$ in pericytes),[39] and cultured human brain pericytes have been shown to express AT1 receptors[40] (transcriptome data[13] also show AT1R expression at the mRNA level in brain pericytes). The transience of the constriction might reflect receptor desensitisation[41] at this relatively high angiotensin II concentration, or a delayed activation of Mas receptors after the angiotensin II is converted to angiotensin (1-7). Blocking either AT2 receptors (with 1 μM PD123319) or Mas receptors (with 10 μM A779) increased the angiotensin II evoked constriction (approximately 4.5-fold for MasR block, $p<10^{-4}$, Fig. 1G-H), consistent with the AT1R-mediated constriction being opposed by angiotensin II activating AT2 receptors, by angiotensin-(1-7) activating Mas receptors, or by activation of AT2/Mas heteromeric[42] receptors.

***SARS-CoV-2 binding potentiates angiotensin II evoked capillary constriction***

Acute application of the RBD of Covid 19 (at 0.7 mg/l, or ~22.5 nM, which is approximately 5 times the $EC_{50}$ for binding)[43] for up to 40 mins evoked a small and statistically insignificant reduction of capillary diameter at pericytes (Fig. 2A). On applying a very high level of angiotensin II (2 μM) in the absence of RBD, a transient constriction of capillaries at pericytes was observed (6.3±3.6% in 6 capillaries, not significantly different from the 7.5±1.6% observed using 150 nM angiotensin II in 9 capillaries in Fig. 1F, p=0.73). However, if brain slices were exposed for 30 min to RBD (0.7 mg/l) before the same concentration of angiotensin II was applied together with the RBD, then the angiotensin II evoked a 5-fold larger constriction of 31.5±9.3% in 4 capillaries (significantly different to that seen in the absence of RBD, p=0.019, Fig. 2B). The 30 min pre-exposure period was used in order to allow time for the large RBD molecule to diffuse into the slice, and was mimicked for the experiments without the RBD. This large constriction-potentiating effect of the RBD was not a non-specific effect on the pericytes' contractile apparatus, because the contractile response to U46619 (200 nM) was unaffected by the RBD (Fig. 2C), and is consistent with the RBD reducing ACE2 activity and decreasing generation of the MasR-activating vasodilator angiotensin-(1-7).

The high concentration of angiotensin II used in Fig. 2B is probably unphysiological and evokes a transient response for reasons that are discussed above. We therefore switched to a lower angiotensin II concentration (50 nM, Fig. 2D), which is more similar to levels that have been found physiologically within the kidney[44,45] and heart.[46] In the presence of the RBD, the constricting response to angiotensin II was increased from an insignificant dilation of 4.5±3.0% to a constriction of 7.8±3.6% (9 capillaries each, p=0.02), i.e. effectively a constriction of ~12% (from 100*{1 - (92.2%/104.5%)}).

Using surface plasmon resonance to assess binding of RBD mutants to immobilised ACE2, we identified the Y489R mutation as reducing binding by ~94% (Fig. 2E). Applying this mutated RBD (for which glycosylation of the protein is expected to be the same as for the normal RBD) had essentially no effect on the response to angiotensin II (Fig. 2D, F). Thus, the potentiation of the angiotensin II response by the RBD is a result of it binding to ACE2.

***The RBD effect is mimicked by blocking ACE2, and blocked by losartan***

We hypothesised that the potentiating effect of the RBD on the response to angiotensin II reflects a decrease in the conversion by ACE2 of vasoconstricting angiotensin II into vasodilating angiotensin-(1-7). Such a decrease is expected if RBD binding promotes ACE2 internalisation[6,8] or if it occludes the angiotensin II binding site. We therefore tested the effect of an ACE2 inhibitor (MLN-4760, 1 μM)[47] on the response to 50 nM angiotensin II. This closely mimicked the potentiating effect of the RBD, confirming that the RBD reduces effective ACE2 activity (Fig. 3A, B). Furthermore, applying the ACE2 inhibitor after inducing constriction with angiotensin II in the presence of the RBD evoked no further constriction (Supp. Fig. 2A-B). This occlusion of the potentiation of the constrictions evoked by the RBD and by the ACE2 inhibitor is consistent with the effect of the RBD being to effectively decrease ACE2 activity.

Activating the Mas receptor which angiotensin-(1-7) acts on (using the stable angiotensin-(1-7) analogue AVE0991), after the capillaries had been constricted by applying the RBD and angiotensin II, led to a large dilation, resulting in a small net constriction similar to that produced by angiotensin II in the absence of the RBD (Supp. Fig. 2C-D). This is consistent with the large constriction seen in the presence of RBD and angiotensin II being the result of the RBD blocking production of angiotensin-(1-7) by ACE2. Applying the MasR blocker A779 (10 μM) during the constriction evoked by angiotensin in the presence of the RBD evoked no further constriction (Supp. Fig. 2C-D), which is also consistent with the RBD inhibiting the generation of angiotensin-(1-7) by ACE2.

The reduction by the RBD of ACE2 activity may reflect ACE2 removal from the surface membrane, either by internalisation[6,8] or (as seen for the related SARS virus) by cleavage and release into the extracellular solution.[48] To assess this, after 3 hours exposure of brain slices to solution containing or lacking the RBD (0.7 mg/l), we used immunohistochemistry to quantify the amount of ACE2 that remained in the cell membrane (defined by overlap in location with PDGFRβ: Supp. Fig. 3A-D). Incubation with the RBD reduced the surface membrane ACE2 level defined in this way (but not the PDGFRβ level) by 32% ($p<0.0001$, Supp. Fig. 3G-H). This figure is an underestimate because of the limited ability of immunohistochemistry to spatially distinguish ACE2 in the membrane from ACE2 internalised to an intracellular position which may be just under the cell membrane, especially in the processes of the pericytes which are too thin for antibody labelling and light microscopy to resolve any intracellular space with no PDGFRβ labelling (see PDGFRβ labelling of processes in Supp. Fig. 3), indeed the fact that the RBD produces a potentiation of the angiotensin II evoked constriction which is similar to that produced by blocking ACE2 (Fig. 3A-B) implies that essentially all of the ACE2 is removed from the surface membrane Measuring the mean intensity of intracellular ACE2 labelling within pericyte somata (which was not feasible for the fine processes of pericytes) showed that the RBD evoked a reduction of level of 25% ($p=0.002$, Supp. Fig. 3E, F, I). This could reflect an RBD-evoked decrease of ACE2 synthesis and targeting of internalised ACE2 for degradation, or an overall loss of ACE2 from the cell as a result of RBD-evoked cleavage and ectodomain release.[48] Crucially, however, the pharmacological data presented in this paper (Figs. 1G, 2D, 3A, 3C, and Supp. Figs. 2A and 2C) imply a loss of functional ACE2 from the outer surface of pericytes and a resulting loss of Mas receptor evoked dilation (opposing AT1R-mediated constriction) in response to angiotensin II.

With a view to reducing SARS-CoV-2 evoked capillary constriction and any associated reduction of microvascular blood flow, we tested whether the AT1 receptor blocker losartan prevented the constriction-potentiating effect of the RBD. Losartan completely blocked the angiotensin II evoked constriction seen in the presence of the RBD (Fig. 3C, D).

In human SARS-CoV-2 infection it has been suggested that one pathological mechanism is a loss of pericytes caused by viral infection reducing their viability or their interactions with endothelial cells.[49] In a transgenic model of pericyte loss (decreasing PDGFRβ signalling) it was found that endothelial cells upregulated von Willebrand Factor (vWF) production, and thus produced a pro-thrombotic state, which could explain the coagulopathy seen in SARS-CoV-2 patients.[49] However, exposing hamster brain slices to RBD (0.7 mg/l) for 3 hours, in the absence or presence of 50 nM angiotensin II, had no significant effect on pericyte death as assessed by propidium labelling (Fig. 4A). Nevertheless, infection with the actual virus might have more profound effects on pericyte function or viability than does exposure to the RBD.

***Capillary constriction is potentiated by SARS-CoV-2 RBD in human capillaries***

To assess whether the potentiation of capillary constriction, characterised above in hamsters, also occurs in human capillaries, we employed brain slices made from live human brain tissue that was removed in the course of tumour removal surgery.[15] Consistent with the similar binding[33] of the SARS-CoV-2 RBD to human and hamster ACE2, we found that the RBD greatly potentiated the pericyte-mediated constriction evoked in human capillaries by 50 nM angiotensin II (Fig. 4B-C). SARS-CoV-2 binding would therefore be expected to decrease human cerebral blood flow assuming that, as in rodents, the largest resistance to flow within the brain parenchyma is provided by capillaries.[50-52]

***Pseudovirus expressing SARS-CoV-2 spike protein evokes capillary constriction***

To check whether a viral stimulus more realistic than the RBD alone would also evoke pericyte-mediated capillary constriction, we constructed SARS-CoV-2 spike protein pseudotyped non-replicating HIV-1 virions (as previously described,[31] see Materials and Methods). After pre-incubating hamster brain slices with these virions, applying 50 nM angiotensin II evoked a constriction of capillaries at pericyte somata of ~11%, compared to the diameter seen in the absence of the virions (Fig. 4D). This is remarkably similar to the potentiated constriction seen when applying the RBD in Figs. 2D and 4B. Plotting the capillary diameter as a function of distance from the pericyte somata (Fig. 4E) showed that the diameter at the soma was larger than that at a distance 10-15 μm from the soma in the absence of the virions, but was smaller than that at a distance of 10-15 μm in the presence of the virions. A similar variation of diameter with distance in the presence of a constricting agent has previously been shown to be consistent with the distribution of circumferential processes as a function of distance from the pericyte soma.[15]

## Discussion

The data presented above are consistent with the scheme shown in Fig. 4F-G. ACE2 expression in the brain appears to be largely on pericytes in both rodents[10] and humans[11] (some papers[53,54] which did not use pericyte or vascular markers have also reported it on endothelial cells, neurons and astrocytes: however, endothelial and astrocyte labelling at the RNA level could reflect contamination[10] with fragments of pericytes or smooth muscle cells). Binding of the SARS-CoV-

2 RBD to ACE2 in pericytes leads to a decrease in effective surface membrane ACE2 activity, which could occur either as a result of ACE2 removal from the membrane (via internalisation[6,8], or cleavage and release into the extracellular solution)[48] or due to occlusion of the angiotensin II binding site (we favour removal as the mechanism, because we detect a decrease in the amount of ACE2 in the surface membrane (Supp. Fig. 3G) and because it is known that, for both the related SARS virus[22] and for SARS-CoV-2,[23,24] binding to ACE2 does not occlude the binding site for angiotensin II). This loss of ACE2 function leads to an increase in the local concentration of vasoconstricting angiotensin II and a decrease in the concentration of vasodilating angiotensin-(1-7) (note, however, that this postulated mechanism is based on the pharmacological experiments reported in Figs. 1G, 2D, 3A, 3C, and Supp. Figs. 2A and 2C, and not on direct measurements of peptide concentrations, the local values of which at pericytes may not be reflected in the bulk concentrations in the solution perfusing the slice). The resulting activation of contraction via AT1 receptors in capillary pericytes reduces capillary diameter locally by ~12% when 50 nM angiotensin II is present. As most of the vascular resistance within the brain parenchyma is located in capillaries.[50-52] this could significantly reduce cerebral blood flow (as occurs following pericyte-mediated constriction after stroke and in Alzheimer's disease).[14,15] In addition, constriction of some capillaries but not others can lead to tissue hypoxia even without a large reduction of blood flow.[55,56] Presumably the same mechanisms could evoke a similar reduction of blood flow and oxygen delivery in other organs where pericytes (or other nearby cells) express ACE2 and AT1 receptors.

We have assumed in this discussion that the AT1 receptors and ACE2 that mediate SARS-CoV-2 evoked constriction are both located on pericytes. However, AT1 receptors are also expressed on other cell types (Supp. Fig. 1A-B) and, although a direct pericyte contractility-regulating effect of angiotensin seems likely, we cannot rule out an indirect effect mediated by AT1Rs on another cell type. Furthermore, even if the AT1Rs are located on pericytes, it may not even be necessary for the ACE2 which is effectively inactivated by SARS-CoV-2 to be located on the same pericytes: depending on how far the angiotensin(1-7) made by ACE2 can diffuse (i.e. how local its actions are), it is conceivable that removal of ACE2 from the membrane of other cells close to pericytes could also promote the vasoconstricting action of angiotensin II on the pericytes.

Constriction of capillaries by pericytes decreases cerebral blood flow in three ways. First, the reduction of capillary diameter increases the local flow resistance because, by Poiseuille's law, resistance to the flow of a liquid is inversely proportional to the 4th power of diameter (e.g. if the 12% diameter reduction mentioned above occurred uniformly in the vasculature then the blood flow would be reduced by 40% [from $(1-0.12)^4 = 0.6$], however pericytes occur only every 30-100 μm (depending on age) along capillaries, implying a less profound effect on resistance). Secondly, the presence of red blood cells results in the blood viscosity increasing dramatically at small diameters,[57] so that even small pericyte-mediated constrictions can have a large effect. Thirdly, complete stalling of blood flow in capillaries can occur as a result of neutrophils (which are less distensible than red blood cells) becoming stuck at narrow parts of the vessel, for example near constricted pericytes,[58-61] and this also increases the reduction of blood flow produced by a small constriction. In the Supplementary Information we estimate that the first two of these factors would reduce overall flow by ~16%, to which neutrophil block may add[58] another 5%. A combined reduction of cerebral blood flow by ~20% is expected to lead to cognitive impairment such as an inability to maintain attention, and white matter damage.[62-64] How long this reduction of blood flow lasts may depend on the time needed for the surface membrane ACE2 level to recover after SARS-CoV-2 infection, which may in turn depend on whether long-term damage is evoked in pericytes.

In order for SARS-CoV-2 to evoke pericyte-mediated capillary constriction (or to cause pericyte dysfunction that upregulates vWF production)[49] the virus would need to bind to the ACE2 that is located in pericytes located on the opposite side of the endothelial cell barrier from the blood. Infection of brain pericytes by SARS-CoV-2 has been reported,[11] raising the question of how the virus can access the pericytes. This might occur via initial infection of the nasal mucosa and movement from there up the olfactory nerve into the brain.[65,66] Alternatively, movement of the S1 part of the Spike protein across the blood-brain barrier by transcytosis has been reported,[67] and crossing the endothelial cell layer may also occur via infection of monocytes (which express ACE2 highly[68] and can cross endothelial cells), or via breakdown of the blood-brain barrier as a result of cytokines released as a result of lung inflammation.[69]

The reduction of blood flow produced by pericyte-mediated capillary constriction, together with any upregulation of vWF that may occur,[49] will tend to promote clotting in the microvasculature. SARS-CoV-2 infection is associated with thrombus formation[70] in large vessels that can be imaged, but it seems possible that thrombi of microvascular origin[71] may add to this, and could perhaps even seed these larger clots. Together, capillary constriction and thrombus formation will reduce the energy supply to the brain and other organs, initiating deleterious changes that probably contribute to the long duration symptoms[72] of “long Covid”. Indeed, the decrease of cerebral blood flow occurring during SARS-CoV-2 infection[18,19] outlasts the acute symptoms.[20] Our data suggest an obvious therapeutic approach, i.e. that the reduction of cerebral and renal blood flow that is observed in SARS-CoV-2 infection[18-21] might be blockable using an AT1 receptor blocker such as losartan. A small clinical trial of the possible beneficial effects of losartan in SARS-CoV-2 infection reported no effect on hospitalisation rate,[73] but did not assess effects on organ blood flow nor long-term outcome such as the incidence of “long Covid”. In contrast, a retrospective study[74] concluded that angiotensin receptor blockers had beneficial effects on clinical outcome in Covid-19.

## Acknowledgements

We thank Lorena Arancibia-Cárcamo for help with microscopy.

## Funding

Supported by a HRH Princess Chulabhorn College of Medical Science Scholarships to CH and JK, MRC CARP award (MR/T005297/1) to GJ, Rosetrees Trust grant (CF1/100004) to DA and FF, BBSRC grant (BB/S017496/1) to JTK, EPSRC grant (EP/S025243/1) to RJO and JH, MRC Career Development Award (MR/R008698/1) to LEM, and ERC (BrainEnergy) and Wellcome Trust Investigator Awards (219366/Z/19/Z) to DA. RBD and mutant RBD protein were provided by the UK COVID-19 Protein Production Consortium.

## Competing interests

The authors report no competing interests.

For the purpose of Open Access, the author has applied a CC BY public copyright licence to any Author Accepted Manuscript version arising from this submission. Source data for the figures are available in a supplementary file associated with this paper.

## Supplementary material

Supplemntary material is availbale at *Brain* online.

### Figure Legends

**Figure 1 Cerebral pericytes express ACE2 and constrict capillaries in response to Ang II.** (**A**) Labelling of hamster cortical slice with antibodies to the SARS-CoV-2 receptor ACE2, the pericyte markers NG2 and PDGFRβ, and with DAPI to label nuclei (**B**) Lower magnification (note different scale bar) maximum intensity projection of ACE2 and PDGFRβ labelling, showing capillaries and penetrating arteriole. (**C**) Integrated ACE2 labelling overlapping with a binarised mask of PDGFRβ labelling and with the inverse of this mask. (**D**) Integrated ACE2 labelling over capillaries versus penetrating arterioles (PA). In (C) and (D) the number of image stacks is on the bars; data in C and D were each from 2 animals. (**E**) Average normalised diameter changes (mean±s.e.m.) at 7 pericytes (in different brain slices from 4 animals) exposed to the thromboxane $A_2$ analogue U46619 (200 nM), and then with the neurotransmitter glutamate (500 μM) superimposed. (**F**) Average normalised diameter changes at 9 pericytes (in different slices from 7 animals) exposed to 150 nM angiotensin II alone (i.e. in artificial cerebrospinal fluid, aCSF), and 10 pericytes (from 3 animals) exposed to angiotensin II in the presence of the AT1R blocker losartan (20 μM). (**G**) As in (F) (aCSF plot is the same) but showing angiotensin II response in the presence of the AT2R blocker PD123319 (1 μM, 9 pericytes from 4 animals) or the Mas receptor blocker A779 (10 μM, 5 pericytes from 2 animals). (**H**) Peak constriction evoked by angiotensin II in different conditions (number of pericytes studied shown on bars). Points superimposed on bar graphs here and in subsequent figures are individual data points (pericytes or image stacks) contributing to the mean.

**Figure 2 The SARS-CoV-2 RBD potentiates angiotensin-evoked capillary constriction.** (**A**) Perfusion of brain slices with RBD (0.7 mg/l) has no significant effect on capillary diameter at pericytes (mean±s.e.m.; n=8 pericytes each for aCSF and RBD, from 3 and 4 animals respectively). (**B**) After preincubation in aCSF for 30 mins, applying 2 μM angiotensin II evokes a small transient capillary constriction at pericytes (n=6, from 3 animals), while including RBD (0.7 mg/l) in the solutions results in an ~5-fold larger response to angiotensin II (n=4 from 2 animals; peak constriction plotted is slightly larger than the mean value quoted in the text because the latter was averaged over 5 frames and here only every 5th frame is plotted). (**C**) RBD has no effect on constriction evoked by 200 nM U46619 (n=6 pericytes for aCSF and 5 for RBD from 2 animals each). (**D**) Response to 50 nM angiotensin II after pre-incubation and subsequent perfusion with aCSF, or aCSF containing RBD or Y489R mutant RBD (n=9 for each, from 3, 4 and 3 animals, respectively). (**E**) Surface plasmon resonance responses for RBD and mutant (Y489R) RBD binding to immobilised human ACE2. (**F**) Mean constriction between t = 29.67 and 30.00 min in (D).

**Figure 3 The effect of RBD is mimicked by blocking ACE2 and reduced by losartan.**
(**A**) Capillary constriction at pericytes in response to 50 nM angiotensin II in the absence (n=9) and presence (n=9) of the RBD (mean±s.e.m., replotted from Fig. 2D) or the presence of the ACE2 inhibitor MLN4760 (1 μM, 9 pericytes from 3 animals, with no RBD). (**B**) Constriction in (A) between t = 29.67 and 30.00 min. (**C**) Response to 50 nM angiotensin II after 30 mins incubation in (and continued perfusion with) aCSF containing RBD (0.7 mg/l, replotted from Fig. 2D) or additionally losartan (20 μM, 10 pericytes from 3 animals). (**D**) Constriction in (C) between t = 29.67 and 30.00 min.

**Figure 4 SARS-CoV-2 potentiates constriction in human and hamster capillaries.**
(**A**) Percentage of pericytes dead (assessed with propidium iodide) in hamster brain slices (numbers on bars, from 2 animals) after 3 hours incubation in aCSF, or aCSF containing 50 nM angiotensin II and/or RBD (0.7 mg/l). (**B**) Effect of 50 nM angiotensin on capillary diameter (mean±s.e.m.) at pericytes in human brain slices in the presence (5 pericytes from 2 humans) and absence (4 pericytes from 2 humans) of the RBD (30 mins pre-incubation). (**C**) Mean constriction at 30 mins) from data in (B). (**D-E**) SARS-CoV-2 pseudotyped virus (see Materials and Methods) evokes pericyte-mediated capillary constriction. (**D**) Capillary diameter at hamster cerebral cortex pericyte somata after incubation with angiotensin II alone (305 pericytes from 2 animals) or with pseudotyped virus and angiotensin II (289 pericytes from 2 animals). (**E**) Capillary diameter as a function of distance from pericyte somata in the presence (289-255 pericytes per point from 2 animals) and absence (305-277 pericytes per point from 2 animals) of pseudotyped virus, in both cases with angiotensin II. The pseudotyped virus induces constriction specifically at the somata. (**F-G**) Likely mode of operation of RBD binding to ACE2. (**F**) Normally, angiotensin II (e.g. generated by the brain renin-angiotensin system (RAS)) can act on vasoconstricting AT1 receptors or vasodilating AT2 receptors, and is converted (pink arrow) by pericyte ACE2 to vasodilating angiotensin-(1-7) that acts via vasodilating Mas receptors. (**G**) In the presence of SARS-CoV-2, binding of the Spike protein RBD to ACE2 leads to its internalisation or cleavage and secretion (see main text), reducing the conversion of angiotensin II to angiotensin-(1-7). Angiotensin II (derived from the brain RAS or from the peripheral RAS) will then evoke a different balance of responses via the receptors shown, generating a larger constriction because of less activation of Mas receptors.

**Figure 1**
**159x227 mm ( x DPI)**

**Figure 2**
**159x128 mm ( x DPI)**

**Figure 3**
**159x143 mm ( x DPI)**

**Figure 4**
**159x198 mm ( x DPI)**

## Supplementary Material

awac272_Supplementary_Data

