# Supplementary material for: SARS-CoV-2 triggers pericyte-mediated cerebral capillary constriction
Source: Brain. 2022 Jul 22;146(2):727–38. doi: 10.1093/brain/awac272 (PMC9384509; doi:10.1093/brain/awac272)
Supplement: awac272_Supplementary_Data [file awac272_supplementary_data.pdf]

**Supplementary Material**

## Estimation of cerebral blood flow decrease evoked by pericyte constriction

This derivation follows that presented previously,[15] but with the addition of the dependence[57] of blood viscosity ($\eta$) on capillary diameter (d). For diameters between 3 and 8 $\mu$m, as are relevant here, the relative viscosity of the blood ($\eta$) is increased at small diameters by a factor given by equations (6) and (7) of ref. 57. For a haematocrit of 0.45, best fitting an inverse power dependence on capillary diameter to this dependence shows that it can be approximated by a relationship that is inversely proportional to $d^N$, where N=1.647 (see Supplementary Figure 4).

We assume that 2L is the distance between pericyte somata, and that the RBD-induced pericyte contraction reduces the capillary diameter from a value of $d_1$ at the midpoint between pericytes to $d_2$ near the pericyte soma (because more circumferential contractile processes are present near the pericyte soma; see Figs. S2 and S3 of ref 15). If, for simplicity, this reduction is linear with distance, the resistance of the capillary segment from the soma to the midpoint is given by

$$k \int_0^L \eta(d).(1/d^4)\, dx$$

where k is a constant, $d(x)=d_2+(d_1-d_2)(x/L)$, the integral is from the pericyte soma (x=0) to half the distance between pericytes (x=L), $\eta(d)$ is the relative viscosity as a function of the diameter at point x (proportional to $1/d^N$ as above), and $1/d^4$ is the Poiseuille dependence of resistance on diameter. For $\eta(d)$ proportional to $1/d^{1.647}$ as above, this integral becomes

$$(k.L/(3+N)).(d_1^{(3+N)}-d_2^{(3+N)})/[d_1^{(3+N)}.d_2^{(3+N)}.(d_1-d_2)].$$

As $d_2$ approaches $d_1$ this approaches

$$k.L/d_1^{(4+N)}$$

so, relative to the resistance when the diameter is uniform at $d_1$, the resistance with $d_2$ different to $d_1$ is

$$[(d_1/d_2)^{(3+N)}-1]/[(1-(d_2/d_1)).(3+N)],$$

where N=1.647 as above.

Inserting the measured changes of diameter from Fig. 2D when applying angiotensin II in the absence or presence of RBD (a 4.5% dilation or 7.8% constriction at the soma, respectively, superimposed on an approximately 10% dilation seen at the soma in the absence of applied drugs[15]) leads to a prediction that the capillaries will increase in resistance by a factor of 40%. Taking into account the fact that the capillary bed provides 57% of the total resistance of arterioles, capillaries and venules[51] (for blood flowing to layer 4 of the cortex) this leads to a prediction that cerebral blood flow will be reduced by ~16% by the RBD. This derivation is oversimplified in that it:

(i) assumes that all pericytes constrict by the same amount, whereas mid-capillary bed pericytes may affect blood flow less[14] than those on the first few capillary branches from the penetrating arteriole (though even mid capillary bed pericytes do regulate[75] blood flow);

(ii) assumes a linear change of diameter with position from pericyte somata to a position midway between adjacent pericytes.

On top of this predicted 16% flow reduction, blockage of capillaries by neutrophils, probably at pericytes, may add another 5% reduction.[58] Thus, the RBD-potentiated angiotensin II evoked constriction might reduce cerebral blood flow by ~20%.

## Supplementary Figure Legends

**Supplementary Figure 1. Labelling of AT1 receptors in hamster cortical pericytes.** (**A**) Low magnification views of cortical labelling, with and without the primary antibodies for AT1Rs and PDGFRβ present, showing that AT1R is expressed on many cells and that omitting the primary antibody abolishes the labelling. (**B**) Labelling of a pericyte for PDGFRβ, DAPI and AT1Rs, showing the soma (white arrow head), pericyte processes running longitudinally along (L) and circumferentially around (C) the capillary, PDGFRβ present in the cell membrane and AT1R expression. (**C**) The capillary bed in hamster cortex, showing pericytes on several vessels, with IB4 surrounding the cell, including on the side of the cell away from the endothelial cells (white arrow heads) thus distinguishing[29,30] these pericytes from fibroblasts. Dashed white lines are to indicate position of capillary in the AT1R image.

**Supplementary Figure 2. The RBD is inferred to reduce ACE2 surface membrane expression.** (**A**) After inducing a potentiated constriction with the RBD and angiotensin II, superimposing the ACE2 blocker MLN4760 induces no extra constriction (9 pericytes from 2 animals). (**B**) Quantification of the constriction at 10 min (Con) and at 30 min (MLN4760) in (A). (**C**) After inducing a potentiated constriction with the RBD and angiotensin II, superimposing the Ang-(1-7) analogue AVE0991 to activate Mas receptors returns the diameter to approximately the value it would have had if the RBD had not decreased Ang-(1-7) production by ACE2 (5 pericytes from 2 animals), while the MasR blocker A779 induces no extra constriction (4 pericytes from 2 animals). (**D**) Quantification of the constriction at 10 min (Con) and at 30 min (AVE0991 and A779) in (C).

**Supplementary Figure 3. Assessment of ACE2 level in the surface membrane of pericytes.** (**A**) Composite image of pericyte showing labelling for the surface membrane growth factor receptor PDGFRβ, the enzyme and SARS-CoV-2 receptor ACE2, and the

nucleus (DAPI). (**B**) PDGFRβ labelling alone. (**C**) PDGFRβ labelling automatically segmented in ImageJ (yellow lines) to define the surface membrane of the cell. (**D**) ACE2 labelling with the mask generated in (C) superimposed. (**E**) PDGFRβ labelling with the inner line of the soma membrane mask from (C) superimposed to define the intracellular area within the soma membrane. (**F**) ACE2 labelling as for (E). (**G**) Mean ACE2 labelling intensity in the membrane defined as in (D) is lower in the presence than in the absence (aCSF) of RBD. (**H**) PDGFRβ labelling intensity in the membrane is unaffected by the presence of RBD. (**I**) Mean ACE2 labelling intensity within the soma intracellular space is reduced by the presence of RBD. All panels used 60 pericytes from 2 animals for each condition (aCSF and RBD).

**Supplementary Figure 4. Dependence of relative blood viscosity on capillary diameter.** Blue curve shows dependence of relative blood viscosity ($\eta$) on capillary diameter for a haematocrit of 0.45, from eqns (6) and (7) of ref. 57. Black curve shows a power law best fit approximation to this, with $\eta \alpha 1/D^{1.647}$.

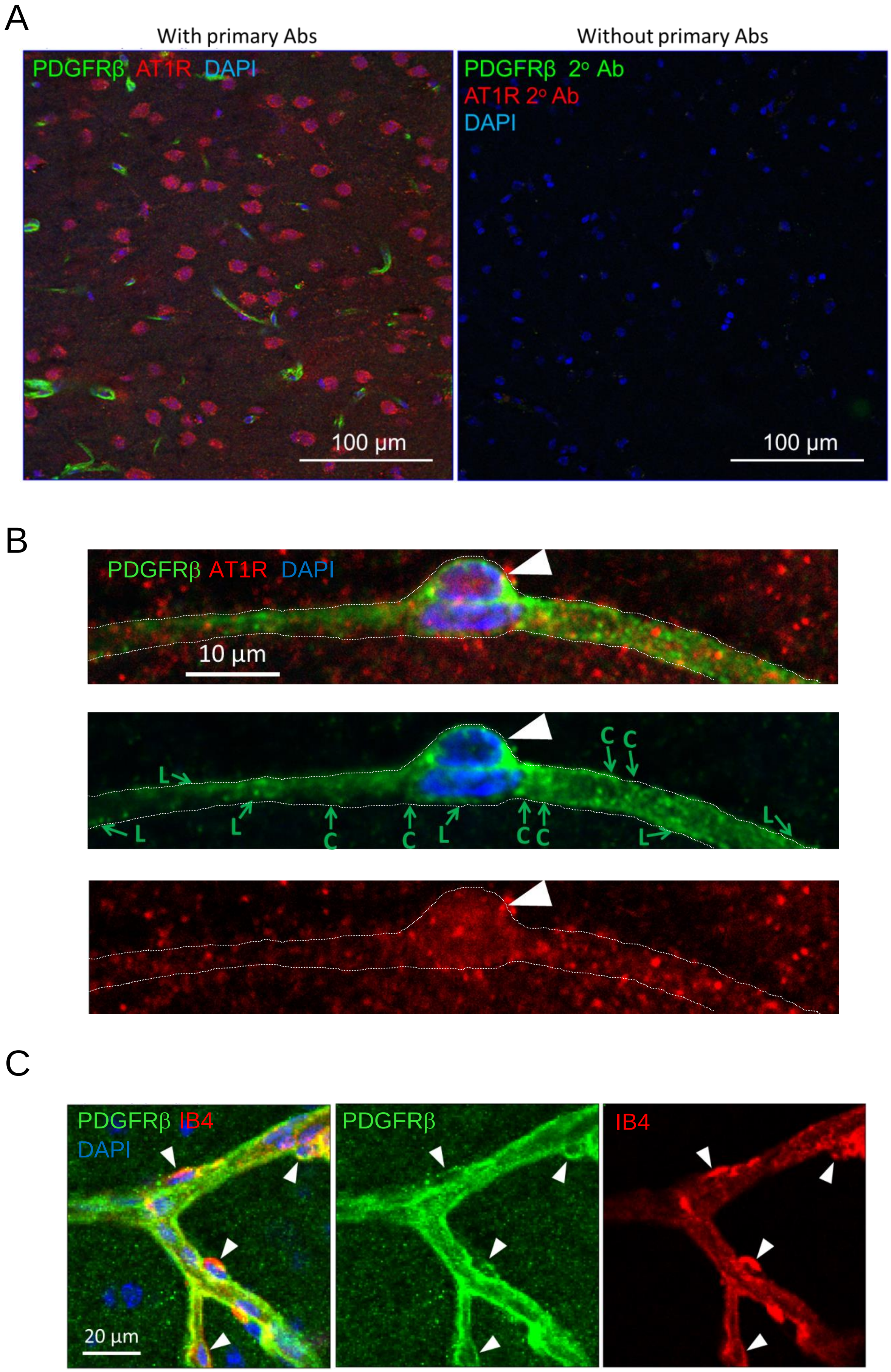


Supplementary Fig. 1

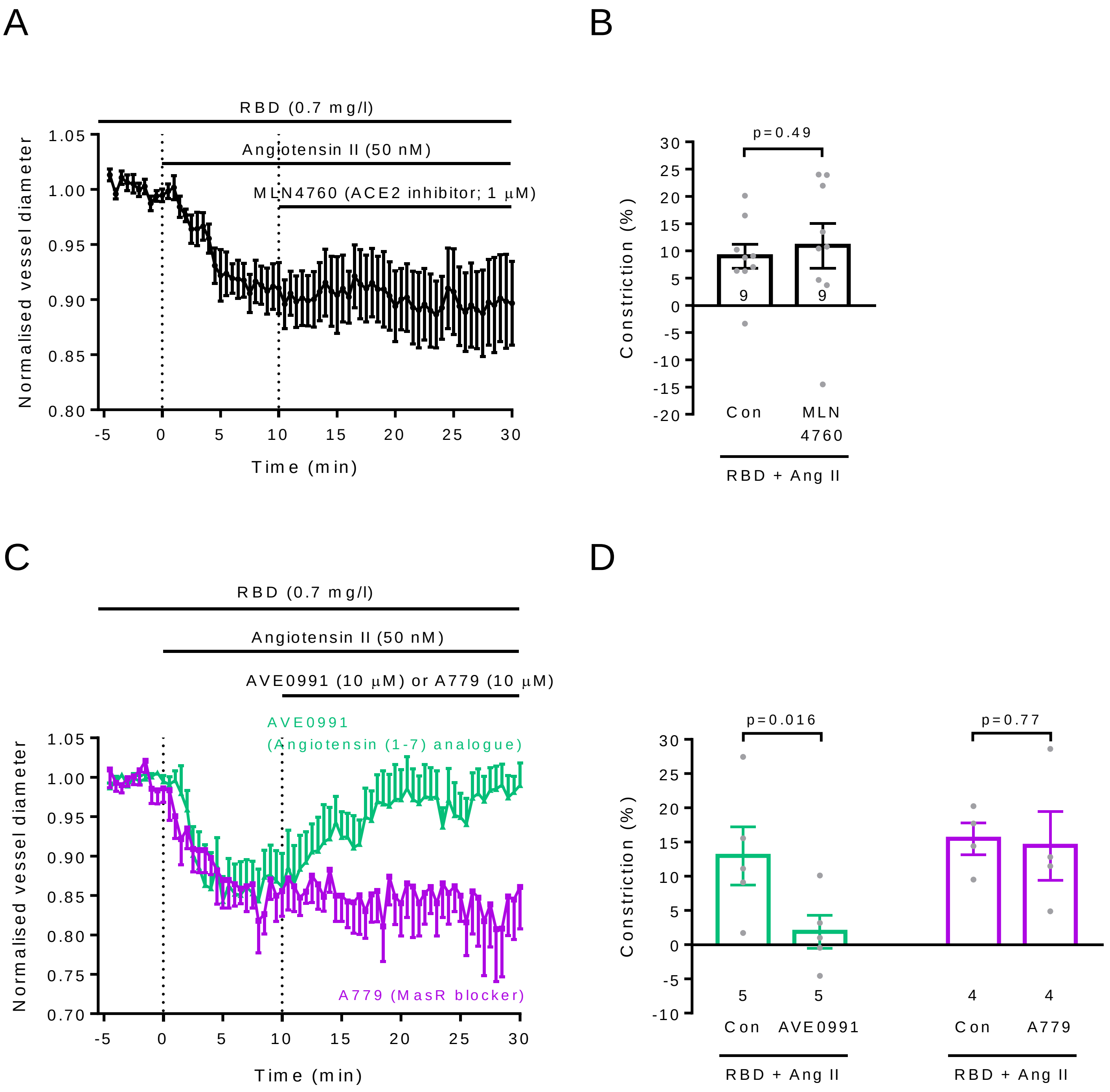


Supplementary Fig. 2

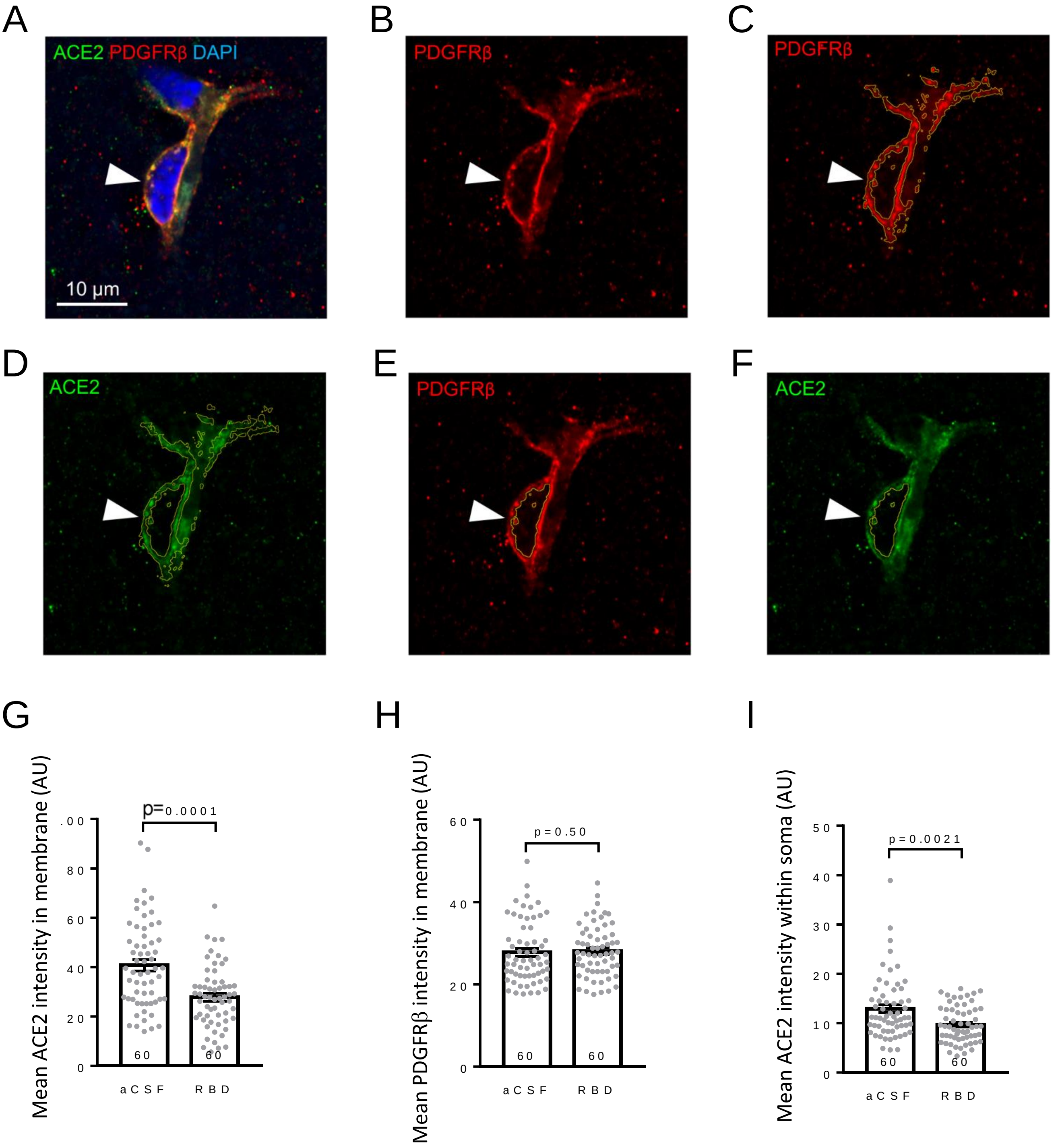


Supplementary Fig. 3

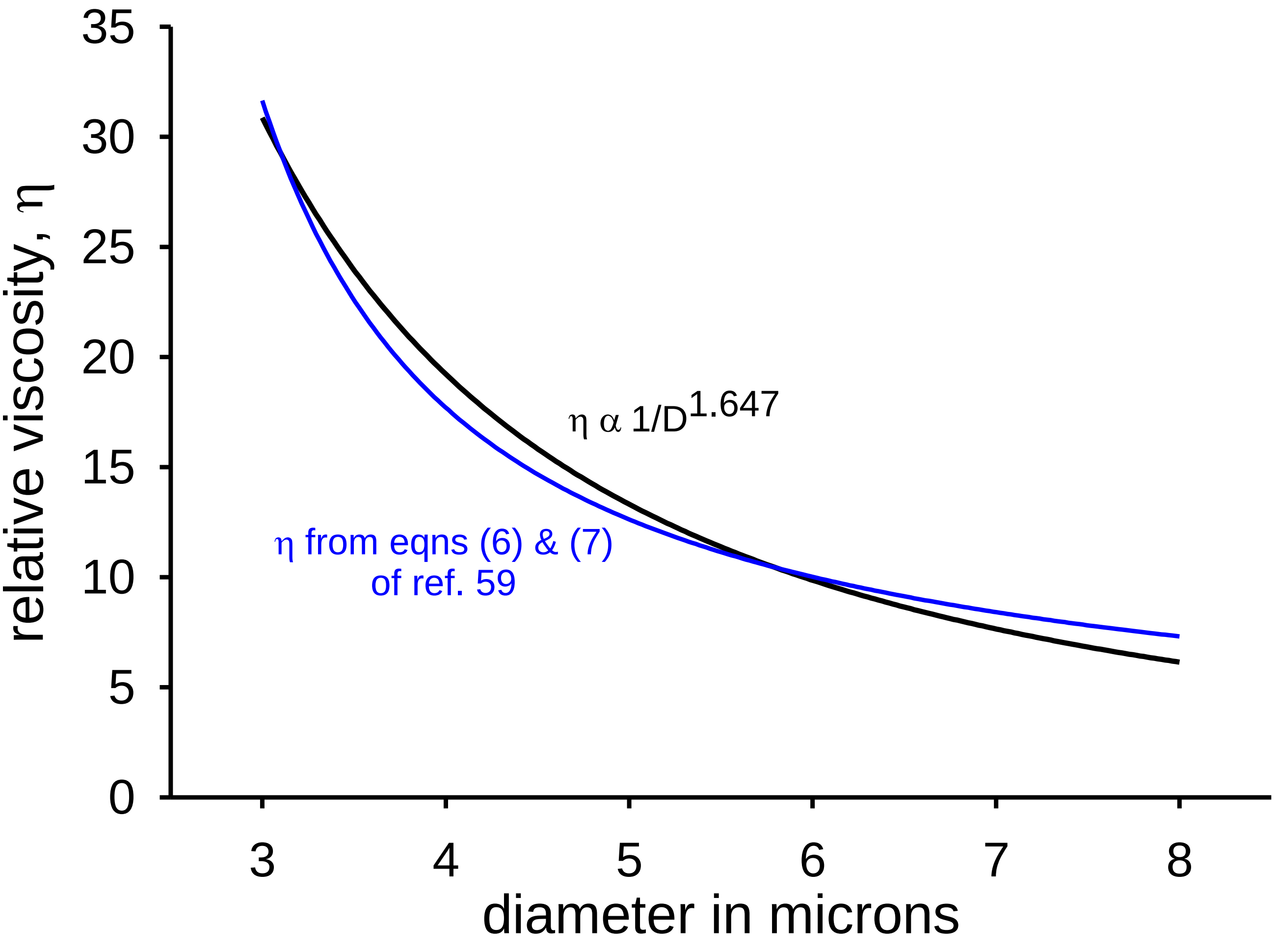


Supplementary Fig. 4
